# A scoping review examining patient experience and what matters to people experiencing homelessness when seeking healthcare

**DOI:** 10.1186/s12913-024-10971-8

**Published:** 2024-04-20

**Authors:** Jean-Philippe Miller, Jennie Hutton, Claire Doherty, Shannen Vallesi, Jane Currie, Katrina Rushworth, Matthew Larkin, Matthew Scott, James Morrow, Lisa Wood

**Affiliations:** 1 St Vincent’s Hospital Melbourne, Melbourne, Australia; 2 The University of Melbourne, Melbourne, Australia; 3 The University of Notre Dame Australia, Perth, Australia; 4 Queensland University of Technology, Brisbane, Australia; 5 St Vincent’s Hospital Sydney, Sydney, Australia; 6 Lived Experience Representative, Melbourne, Australia; 7 Victorian Virtual Emergency Department, Northern Hospital, Melbourne, Australia; 8 School of Psychology and Public Health, La Trobe University, Melbourne, Australia

**Keywords:** Homeless, Patient experience, Health services, Healthcare, Access to care, Outcome measures, Surveys, Scoping review

## Abstract

**Background:**

Homelessness is associated with significant health disparities. Conventional health services often fail to address the unique needs and lived experience of homeless individuals and fail to include participatory design when planning health services. This scoping review aimed to examine areas of patient experience that are most frequently reported by people experiencing homelessness when seeking and receiving healthcare, and to identify existing surveys used to measure patient experience for this cohort.

**Methods:**

A scoping review was undertaken reported according to the PRISMA-ScR 2020 Statement. Databases were searched on 1 December 2022: MEDLINE, EMBASE, APA PsychINFO and CINAHL. Included studies focused on people experiencing homelessness, healthcare services and patient experience, primary research, published in English from 2010. Qualitative papers and findings were extracted and synthesized against a modified framework based on the National Institute for Health and Care Excellence guidelines for care for people experiencing homelessness, the Institute of Medicine Framework and Lachman’s multidimensional quality model. People with lived experience of homelessness were employed as part of the research team.

**Results:**

Thirty-two studies were included. Of these, 22 were qualitative, seven quantitative and three mixed methods, from the United States of America (*n* = 17), United Kingdom (*n* = 5), Australia (*n* = 5) and Canada (*n* = 4). Health services ranged from primary healthcare to outpatient management, acute care, emergency care and hospital based healthcare. In qualitative papers, the domains of ‘accessible and timely’, ‘person-centred’, and values of ‘dignity and respect’ and ‘kindness with compassion’ were most prevalent. Among the three patient experience surveys identified, ‘accessible and timely’ and ‘person-centred’ were the most frequent domains. The least frequently highlighted domains and values were ‘equitable’ and ‘holistic’. No questions addressed the ‘safety’ domain.

**Conclusions:**

The Primary Care Quality-Homeless questionnaire best reflected the priorities for healthcare provision that were highlighted in the qualitative studies of people experiencing homelessness. The most frequently cited domains and values that people experiencing homelessness expressed as important when seeking healthcare were reflected in each of the three survey tools to varying degrees. Findings suggest that the principles of ‘Kindness and compassion’ require further emphasis when seeking feedback on healthcare experiences and the domains of ‘safety’, ‘equitable’, and ‘efficiency’ are not adequately represented in existing patient experience surveys.

**Supplementary Information:**

The online version contains supplementary material available at 10.1186/s12913-024-10971-8.

## Background

Homelessness is associated with large disparities in health, including a much higher prevalence of both chronic conditions and acute illness and injury [1–3]. This perpetuates disproportionate rates of unplanned hospital use [4] and a three-decade gap in life expectancy [5, 6]. Despite significant health needs, people experiencing homelessness (PEH) face numerous barriers to accessing health services and preventive healthcare [7, 8] and are more likely to seek emergency or unplanned healthcare [4, 9]. This is often at a later stage of ill health, leading to lengthy and costly hospital admissions [7]. Individual and structural factors associated with homelessness impact peoples’ capacity to attend appointments, advocate for the support they need and maintain regular contact with health providers that is necessary to improve their health and wellbeing. Among PEH, structural barriers and discrimination are ubiquitous experiences when accessing healthcare [8, 10]. Current evidence associates the experience of stigma for PEH with the perpetuation of existing health inequalities, service avoidance, and subsequent poorer physical and mental health [10–12]. The anachronistic and hierarchical design of many conventional health services is counterproductive to the required trauma-informed approach that facilitates PEH or those experiencing marginalisation and vulnerability to access healthcare when they need it. It is clear that the traditional approach to designing healthcare services could be much improved if PEH were engaged in the process and their voices prioritised”.

Continual improvement of healthcare informed by patient experience is critical for all populations, particularly for PEH given the substantial health disparities and known barriers to healthcare access and engagement. In an effort to tackle health inequities for PEH, health systems must continuously monitor and improve the quality of healthcare they provide [8]. Capturing patient experience across healthcare settings is paramount to drive service improvement [13] and promote more equitable access, especially among PEH. In 2001, the Institute of Medicine (IOM) conceptualised quality principles across six dimensions for improvement (safe, effectiveness, patient-centred, timely, efficient and equitable) in an effort to raise the quality of health care [14]. Measurements of quality healthcare have previously focused heavily on access, clinical care processes, disease-specific indicators and mortality [15]. As part of contemporary healthcare delivery, patients’ experiences of healthcare are considered an indicator of the quality of care [15]. Patient experience is central to improvements in the provision of quality health care [13, 14, 16] and has been positively associated with patient safety and clinical effectiveness [13, 17], higher levels of treatment adherence and less healthcare utilisation [13, 17]. Patient experience is distinct from patient satisfaction in that it asks about the person’s experience of healthcare rather than simply whether they were satisfied or not. This experience-focused feedback provides valuable insights into the quality of care provided and is fed back to providers [16]. Experiences may differ according to the vulnerability of population groups and patient expectations [16]. Surveys and reports on patient experience provide a means of intrinsically evaluating and measuring aspects of care quality from the patient’s perspective, principally offering healthcare services an opportunity to capture and appraise ‘patient-centred care’ as a domain of quality [14, 15]. Patient-centred care is key to the provision of quality health care [17] and has been highlighted as a priority for homeless healthcare [8].

## Methods

### Aim

The aim of this scoping review was to examine the areas of patient experience that are most frequently reported by PEH when seeking healthcare, and how the patient experience for people experiencing homelessness is represented and discussed in the literature and what deficits exist. A secondary objective of this review is to understand what surveys, or components of surveys, are being used to ask about patient experience for PEH.

### Study context and rationale

The study was undertaken in Australia to inform the development of evidence-based strategies for homeless health services. In Australia, the number of PEH is rising [18]. In 2021, there were over 122,000 estimated PEH on any given night, an increase of more than five percent over five years [18]. Existing structured patient-reported experience survey methods have limited applicability for vulnerable populations in Australian hospitals [19]. For example, the Australian Hospital Patient Experience Question Set (AHPEQS) [20], does not adequately report the perspectives of people with low health literacy – a population that is over-represented among PEH [21]. Furthermore, patient experience measures in Australian Primary Healthcare (PHC) settings are not well established, nor standardised [22] and therefore little is known about the accessibility or experiences of these services for PEH. The impetus for this study was thus to identify the ways in which patient experiences of healthcare for PEH has been measured or captured in the international literature to inform the development of homeless healthcare services at the organisation.

### Search strategy

A scoping review was undertaken to explore the broad research aim reported here using the Preferred Reporting Items for Systematic Reviews and Meta-Analyses extension for Scoping Reviews (PRISMA-ScR) [23]. The search strategy was identified and refined by the authorship team, comprising health service managers, academics, people with lived experience of homelessness and clinicians, through an iterative process. Two of the authors had significant experience in providing healthcare to PEH, and three were experienced researchers in homeless health. A librarian was engaged to assist with the initial identification of search terms. The PICO framework was used to develop the search terms and eligibility criteria, as shown in Table 1. The intervention of interest was patient experience for PEH when seeking and receiving healthcare.

**Table 1:** PICO Framework

	**Inclusion criteria**	**Exclusion criteria**
**Population:**	*People experiencing homelessness*^*a*^	*Vulnerable OR ‘at-risk’ populations*
**Exposure:**	*Healthcare service*	*Exposure not a healthcare setting*
**Outcome:**	*Patient experience/perspective of a healthcare service*	*Outcomes are only from the healthcare provider perspective* *Outcomes are not related to a healthcare service* *Outcomes are not patient reported outcomes* *Specialised health service or disease focus*
	*Peer-reviewed primary research, case studies, governmental report*	*Publication types: unpublished manuscripts, dissertations, doctoral thesis, editorials, opinion pieces, study protocol, report, conference proceedings/papers/abstracts, letters to the editor, book sections/reviews, systematic reviews, literature reviews*
	*English language, 2010–2022*	*Published prior to 2010* *Not an OECD Country* *Tool/Survey unavailable or not related to patient experience*

^a^Threshold for homelessness was ≥ 50% of sample currently experiencing homelessness

Three preliminary searches were conducted to identify and test search terms. The final search terms were as follows: Patient reported outcome measures OR patient outcome assessment OR patient satisfaction; Health facilities OR health services OR quality of healthcare OR patients OR (health* or hospital* or patient* or outpatient* or emergency department*.ti,ab,kw.) Health services accessibility OR access*.ti,ab,kw. Searches were grouped with the relevant keyword terms of (patient* or outpatient* or inpatient*) OR (consumer* or client* or adult* or people*) and matched with a set of adjectives with a defined adjacency of two in an effort to capture patient experience. The adjectives utilised were experience* or reported* or perspective* or perceive* or feedback* or complaint* or view* or voice* or preference* or satisfaction* or insight*.ti,ab,kw.

The final search was conducted on 2 February 2024 across the following databases: Medical Literature Analysis and Retrieval System Online (MEDLINE), Excerpta Medica Database (Embase), American Psychological Association PsychINFO and Current Index to Nursing and Allied Health Literature (Cinahl). The final search strategy and terms adapted for each database are available (See Additional file 1).

All search results were exported to EndNote (X9.3.3, Clarivate) and uploaded to the online systematic review collaboration software Covidence (Veritas Health Innovation, Melbourne, Australia) (Available at www.covidence.org). Duplicates were automatically discarded. The titles and abstracts were screened independently by two authors. Conflicts were resolved by a third author. Screening of full-text papers was conducted as described above. To ensure alignment with PHC and hospital services, recency of reported experience and the Australian healthcare system, articles focusing on specialised health services/diseases, accessibility, years 2008/2009, and non-OECD countries were excluded. Systematic reviews that were identified during the screening process were reviewed for any eligible references.

### Data items and extraction

Data extraction occurred through three phases. In Phase 1, the primary author extracted the following core characteristics from included papers: author, year, country, title, study design, objective, population, setting, exposure (inclusive of type of health service) and patient experience measure. To ensure the accuracy of this process, an independent review of the extraction was undertaken by two authors.

In Phase 2, the authorship team sought a framework to meaningfully extract and then code the qualitative patient experience data from the included studies and three were identified: the National Institute for Health and Care Excellence (NICE) guidelines for integrated health and social care for people experiencing homelessness [24], the Institute of Medicine (IOM) Framework for Health Care Quality [25], and Lachman’s multidimensional quality model [26]. The NICE guidelines, developed in the United Kingdom, are a comprehensive resource for working with PEH. The IOM framework is a well-established quality framework used in healthcare to align policy and practice. The IOM domains are a set of principles that are used to guide and improve the quality of healthcare delivery. Lachman’s quality framework builds on the existing IOM principles, offering a new and novel means of assessing quality in healthcare. These three frameworks were modified by removing any overlap to form one extraction framework. The authors added the domain of ‘communication’ to address an obvious deficit in existing frameworks as identified in the NICE guidelines [24] and recent literature [27]. See Tables 2 and 3 for established definitions.

**Table 2:** Modified framework of quality domains

Domain of Quality	Definition	Themes				
Safety	*Care should be free from harm, where harm is defined as something one would not accept for oneself or one’s Kin (physical or psychological)*	*Avoiding harm to patients from the care that is intended to help them*	*Trauma-informed*	*Physically and psychologically safe*	*Accountability*	
Effective	*All care follows evidence-based guidelines and standard operating procedures (SOP) where appropriate, with deviation only as per need of the person receiving care*	*Providing services based on scientific knowledge to all who could benefit and refraining from providing services to those not likely to benefit (avoiding underuse and misuse, respectively)*	*Evidence-based decision making*	*Standard operating procedures*		
Person-Centred	*The care a person receives should be filled with kindness, dignity, and respect. People should be seen as a whole and their care must be coproduced. Shared decision-making and self-management are essential*	*Patient-centred care/specific needs and priorities*	*Pay attention to the diverse experience of people using the service*	*Homeless people need more resources? Longer appointments, more targeted service delivery*	*Social capital*	
Accessible & Timely	*There are no delays in receiving care. Universal quality with safe access is the goal*	*Reducing waits and sometimes harmful delays for both those who receive and those who give care*	*Location of services/Physical and organisational*	*Access to services/Barriers to services*	*Flexibility of services*	*Affordable*
Efficient	*Unnecessary care is not provided. All care should have intended benefit*	*Avoiding waste, including waste of equipment, supplies, ideas, and energy*	*Coordination of services (helps individuals connect the dots across multiple providers and settings)*	*Navigation of services*	*Patient -up (also highlighted under communication)*	
Equitable	*Care is of the same quality all the time, no matter who you are and where you require care*	*Providing care that does not vary in quality because of personal characteristics such as gender, ethnicity, geographic location, and socioeconomic status*	*Aim to address health inequalities*	*Consistency in care responses*		

Definitions taken from Lachman’s multidimensional quality model

Themes within boxes shaded in grey were taken from NICE guidelines for integrated health and social care for people experiencing homelessness

**Table 3:** Modified framework of quality core values

Core Values	Definition	Themes				
**Dignity & Respect**	*All views are accepted and respected in decision making*	*Are inclusive*	*Strength based approach*	*Recovery oriented*	*Support people to overcome stigma*	*Confidentiality/Anonymity/Privacy*
**Kindness with Compassion**	*Appreciation of the human side of the person. Patient/Kin are kind to the provider*	*Empathetic and non judgemental*	*Clinicians and non clinicians warm and welcoming*			
**Holistic**	*Care addresses physical needs as well as spirituality and mental wellbeing in an integrated manner*	*Consider using psychologically informed environments and trauma informed care*	*Treats patient as person not disease and integrates care*	*Scope of services*	*Integration of care (brings siloed services together to create a more seamless patient experience)*	
**Partnership and coproduction**	*Be an active partner in designing health. Able to choose where and how to receive care*	*Relationship/Continuous healing relationship* *Continuity of care*	*Person at the centre of control*	*Recognise the value of codesigning and codelivering services with people with lived experience of homelessness, to improve the quality of health and social care*	*Recognise that people experiencing homelessness, especially those with experience of rough sleeping, need services that provide a long-term commitment to care to promote recovery, stability and lasting positive outcomes*	*Support re-engagement*
**Communication**	*The effective exchange of information between healthcare providers and patients, aiming to enhance understanding, decision-making and improved health outcomes*	*Literacy/No Jargon acronyms* *Provide extra support for people with low literacy levels or with speech, language and communication difficulties. Information Sharing.*	*Give people information to access other services* *Awareness of services. Communication* *with Kin*	*Use communication methods based on the person's preferences/* *Consider the persons circumstances & access to phone or internet*	*Send clear information about contacts or appointments and reminders that reach people in time, and follow up people who do not attend*	*Provide translation and interpretation services if needed, ensure written information is available in different formats and languages, easy to read*

Definitions taken from Lachman’s multidimensional quality model, with the exception of the communication definition, which was developed by the authors

Themes within boxes shaded in grey were taken from NICE guidelines for integrated health and social care for people

The findings of the included papers were extracted against the modified framework by six authors (JPM, JH, CD, LW, SV, JC) simultaneously over three meetings. In some instances, the patient-reported outcomes overlapped between the two domains, perhaps indicating the complexity and subjectivity of patient-reported experiences. Final decisions were made by two authors (JM, CD).

In Phase 3, data from quantitative studies that used a survey to measure patient experience were extracted. Two surveys were excluded from the analysis because they did not have patient experience measures [28, 29]. The following data points were extracted from patient experience surveys: 1) survey name, 2) authors utilising survey, 3) number of survey items 4) number of domains (referred to as scales hereafter to ensure differentiation from the term used in the extraction framework), 5) survey setting, 6) survey questions 7) assignment of survey questions to IOM domains and core values. Data were grouped together for surveys appearing in multiple articles. Data points were extracted directly from articles with the exception of data point 7 which was completed by reviewers (See Additional file 2). Four authors individually analysed each survey question and assigned a primary domain or core value to each. Responses were reviewed by all authors, and any disagreements were discussed and resolved through a consensus vote.

## Results

### Study selection

**Fig. 1:**
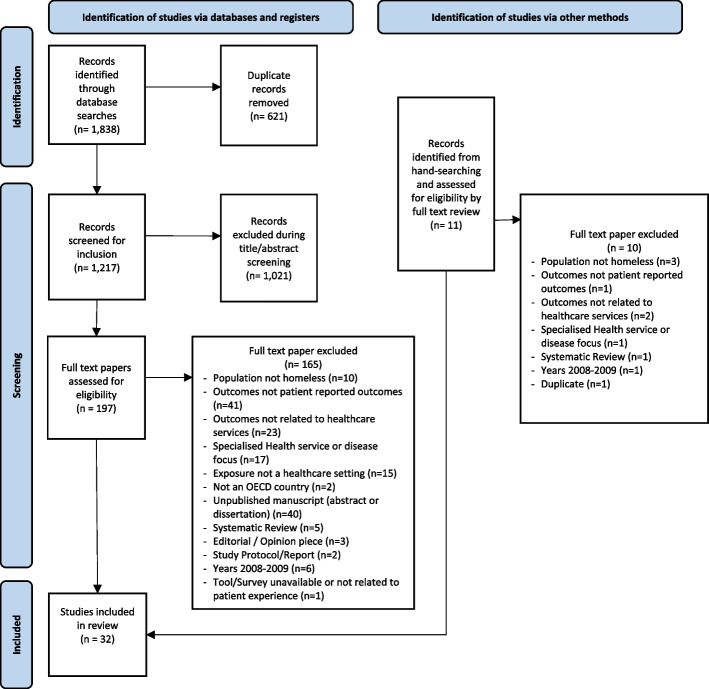
PRISMA flow diagram

One thousand eight hundred thirty-eight records were identified through the database searches, ultimately thirty-two studies were included in this scoping review. Details of the screening process are shown in Fig. 1.

### Study characteristics

Of the 32 included studies, 22 were qualitative, seven were quantitative and three were mixed-method study designs (see Tables 4, 5 and 6). Seventeen studies were from the United States of America (US), five from the United Kingdom (UK), five from Australia and four from Canada. Seventeen studies examined the provision of primary health care, with the remaining studies examining outpatient case management [30], acute care [28], emergency care [31], and hospital-based healthcare [29, 32]. Twelve studies did not define a specific health service context and instead reported information on general patient experiences engaging with any area of health services. The tools utilised across the studies included interviews (*n* = 20), focus groups [29, 31, 33–36] (*n* = 7), surveys [32, 37–43] (*n* = 8), and qualitative ranking exercise [44] (*n* = 1) to facilitate exploration of patient experience. The qualitative ranking exercise asked PEH and experts to prioritise 16 aspects of PHC.

**Table 4:** Qualitative Studies

Author, year Country	Title	Objective	Population/Setting	Exposure	Patient Experience Measure	Key findings related to patient experience measures and/or outcomes
Davis, 2012 [30] US	‘‘Because Somebody Cared about Me. That’s How It Changed Things’’: Homeless, Chronically Ill Patients’ Perspectives on Case Management	To study the perspectives of enrollees in an intensive case management program focused on decreasing admissions among frequently admitted patients at a public hospital in order to understand	PEH *N* = 11 Outpatient intensive case management program	Hospital outpatient case management program	FTF interviews	Participants in the case management program identified their prior social isolation as unhealthy and valued two distinct aspects of the program: feeling cared for through their relationships with case managers and receiving assistance with navigation of medical systems and social services. Participants identified each as important contributors to their improved health
Gunner, 2019 [45] UK	Provision and accessibility of primary healthcare services for people who are homeless: a qualitative study of patient perspectives in the UK	To explore the perspectives of individuals who are homeless on the provision and accessibility of primary healthcare services	PEH *N* = 22 Homeless shelters and a Specialist primary healthcare centre for people who are homeless (SPHCPH)	No specific health service exposure	FTF semi-structured interviews	PEH perceived the model of care at the SPHCPH to be best practice. Mainstream services were associated with negative experiences and inequality in access. Barriers to access highlighted included the denial of registration, fragmented services, poor continuity of care, stigma and discrimination and a lack of awareness on the complexity of healthcare needs for PEH
Henderson, 2022 [33], US	Experiences of Adult Men Who Are Homeless Accessing Care: A Qualitative Study	The purpose of this study was to describe the perceptions of homeless individuals related to their health and experiences accessing care	PEH *N* = 16 Emergency homeless shelter for men	No specific health service exposure	Focus groups *n* = 2	Three themes identified; (1) men who are homeless experience bias throughout their health care and interpersonal relationships; (2) the best care is person-centred and considers patients’ priorities; (3) care coordination resources are inadequate PEH experience access to healthcare as transactional, opting for convenient episodic care as a means of avoiding poor care coordination, power imbalances and stigma. PEH want their priorities to be considered and care to be person-centred. PEH have complex needs and desire assistance in care coordination and navigation of the healthcare system to ensure their needs and priorities can be met
Hirst, 2021 [46], UK	Benefits of GP care in outreach settings for people experiencing homelessness: a qualitative study	To explore PEHs’ experiences of GP care in community outreach settings in UK; and to seek staff/volunteers’ views on the strengths and weaknesses of GP community outreach services	PEH *N* = 22 Drop-in day-centre and Food Drop-in	GP Outreach Service in different community settings Primary Healthcare	FTF semi-structured interviews	Across all settings, GP outreach services helped facilitate access to medical care for PEH. High value was placed on the positive physical, social and organisational environment of GP outreach services. Compared to mainstream services, outreach services were perceived as comfortable, safe, engendered a sense of belonging, convenient and integrated as they brought several services together. PEH valued outreach services as they felt listened to, and time was dedicated to building a therapeutic relationship
Lamanna, 2018 [34], Canada	Promoting continuity of care for homeless adults with unmet health needs: The role of brief interventions	To examine diverse stakeholder perspectives on the role of a brief intervention in supporting continuity of care, using qualitative methods for their strength in studying phenomena that have not been well documented	PEH *N* = 22 People with Lived Experience of Homelessness *n* = 8 Coordinated Access to Care for Homeless People (CATCH) programme	Brief Interventions—CATCH programme Primary Healthcare	Focus groups *n* = 3 Semi structured interviews n = 29	Findings suggest that brief interdisciplinary interventions can promote continuity of care by offering low-barrier access, timely and responsive service provision, including timely connection to long-term services and supports, appropriate individualised services and effective coordination of services. Although brief interdisciplinary interventions were perceived to promote access, timeliness and coordination of care for this population with complex health and social needs, gaps in the local service delivery context can present persisting barriers to care comprehensiveness and continuity
McCallum, 2019 [47], UK	Using always events to derive patient-centred quality improvement priorities in a specialist primary care service providing care to a homeless population	To determine if the Always Events (AE) concept is an acceptable and feasible method for deriving patient-centred QI priorities in a specialist primary care service providing care to PEH	PEH *N* = 20 Drop-in clinics at a specialist homeless general practitioner service	No specific health service exposure Primary Healthcare	Interviews ‘Always Events’ (AE) is a validated QI method	Nine AEs were generated, five fitted the criteria to be used as metrics for future QI projects. These were ‘I always want…’ to be seen, the staff to be approachable and responsive, to feel safe while waiting to be seen, my privacy to be valued and clear information on how service works. The AE method is an acceptable and feasible tool for generating QI targets that can lead to improvements in care for this vulnerable group
Meehan et al. 2023 [48], USA	Previous Health Care Experiences’ Influence on Health Care Perceptions Among Residents in Six Homeless Shelters in Seattle, Washington, July–October 2021	To describe where residents of homeless shelters in Washington receive healthcare, and to examine their perceptions of healthcare and experiences of healthcare	PEH *N* = 68 Participants all residents across 6 homeless shelters	No specific health service exposure	Semi-structured interviews *n* = 25 Focus groups *n* = 8 groups with 43 participants	Findings indicated that residents received healthcare from a variety of settings, with varying frequency. Onsite clinics at homeless shelters and community homeless clinics were most commonly used, but also ED and urgent care and pharmacies Key elements shaping health experiences that emerged from the data were ability access healthcare, level of clear communication from health facilities and staff, ease of securing timely follow up care, experiences of respect or stigma/discrimination. Positive, neutral and negative perceptions of healthcare experiences were identified
Moore, 2011 [49] Australia	Complex health service needs for people who are homeless	To examine the perceptions and experiences of homeless people in relation to their health service needs as well as those of service providers involved with their care	PEH *N* = 20 ED in an acute hospital	No specific health service exposure	Semi structured interviews	Six key themes were identified from interviews: complexity of care needs, respect for homeless people and co-workers, engagement as a key strategy in continued care, lack of after-hour services, lack of appropriate accommodation and complexity of services
Moore-Nadler, 2020 [50] US	Storytelling to capture the health care perspective of people who are homeless	To explore the perceptions of health care experiences by people who are homeless in Mobile, Alabama, while also considering their own thoughts and beliefs regarding the interviews	PEH *N* = 16 Homeless day shelter, Mobile	No specific health service exposure	Semi structured interviews	The following themes were identified: social determinants of health, compromised systems, professionalism, dehumanisation, engagement, and downward trajectory The experiences described and themes identified indicate a breakdown in therapeutic relationships between homeless individuals and health care providers, contributing to the continuing destabilisation common in this population
Nicholas, 2016 [31], Canada	The Experiences and Perceptions of Street-Involved Youth Regarding Emergency Department Services	How do Street Involved (SI) youth experience and navigate ED services? What components of ED services foster accessibility and engagement of SI youth? What components of ED services limit accessibility and engagement of SI youth?	PEH *N* = 42 Health care and service providers in the EDs or community agencies serving SI youth,	Acute and Emergency care	FTF interviews Focus groups *n* = 7	Street Involved (SI) youth often perceived suboptimal care and experienced long waiting periods that led to many avoiding or prematurely exiting the ED. Service gaps appeared to have a negative bearing on their care and health outcomes. Negative interactions and serious health implications periodically left SI youth at heightened risk of long-term and serious health and psychosocial outcomes
Paradis-Gagne, 2023 [51] Canada	Perceptions of mobile and acute healthcare services among people experiencing homelessness	(1) to explore the needs of people experiencing homelessness regarding outreach services and (2) to describe the perceptions and preferences of people who benefit from this outreach intervention	PEH *N* = 12 Mobile clinic outreach service	No specific health service exposure	Semi structured interviews	The core category that emerged from the data analysis was “Perception of Health Care” from the following subcategories: (1) Conflicting Relationships with Institutional Health Services; (2) Perception of Outreach Services; and (3) Recommendations from Mobile Clinic Users. Some PEH were satisfied with the care received in the public health system, while many have experienced dehumanising practices. Outreach services are a promising strategy to reach underserved populations. Findings highlight practices to personalise and adapt healthcare services and foster inclusive environments to better serve PEH
Purkey, 2019 [29], Canada	Experience of healthcare among the homeless and vulnerably housed a qualitative study: opportunities for equity-oriented health care	To explore the experience of hospital-based healthcare for people who are vulnerably housed or homeless	PEH interviews *N* = 31 Agencies providing services to those who are vulnerably housed	Hospital-based Healthcare	Focus groups *n* = 6 Semi structured interviews *n* = 4 (in-person or telephone) Participant and lived experience survey ^**a**^	Four themes were highlighted by participants: (1) experiences and consequences of stigma and shame when accessing healthcare; (2) lack of accountability of the healthcare system towards equity seeking populations; (3) inflexibility of the healthcare system; and (4) positive experiences that warrant discussion for what they teach us about potential improvements
Saharan, 2021 [52], US	Sharing the burden of treatment navigation: social work and the experiences of unhoused women in accessing health services in Santa Cruz	To explores the challenges faced by unhoused women in accessing general and reproductive health care services in Santa Cruz, CA	PEH *N* = 5 Homeless Garden Project	No specific health service exposure	Semi-structured interviews	Women who had access to a social worker were much more likely to report improved access to satisfactory treatment. These findings suggest that there is not a tangible lack of healthcare services for unhoused women in the local community, but rather a burden of treatment navigation caused by a dearth of information on how to access care. The interviews suggest that this burden can be reduced with social work interventions and service centers that offer health navigation support
Steward, 2016 [44], US	Priorities in the primary care of persons experiencing homelessness: convergence and divergence in the views of patients and provider/experts	To identify aspects of primary care important to persons familiar with homelessness based on personal experience or professional commitment, and to highlight where the priorities of patients and professionals dedicated to their care converge or diverge	PEH *N* = 26 Homeless service organisations and established board of homeless service users	No specific Health Service Exposure Primary Healthcare	Qualitative Exercise, card sort	Both groups gave high priority to accessibility, evidence-based care, coordination, and cooperation. Provider/experts endorsed patient control more strongly than patients. Patients ranked shared knowledge and the free flow of information about their care more highly than providers/experts
Strange, 2018 [53], Australia	A general practice street health service: Patient and allied service provider perspectives	To explore patient and staff perspectives of a street-based, primary health service, to help identify factors influencing patient access and management	PEH *N* = 27 Freo Street Doctor (mobile open-access general practice service)	Street based and mainstream primary healthcare Primary Healthcare	Semi-structured interviews	Thematic analysis identified factors influencing patient willingness to access primary healthcare: doctor–patient empathy, better understanding of patient circumstances, fostering of social capital, facilitating referral pathways and supporting the transition to mainstream general practice as circumstances improve. Hospital discharge planning and follow-up were service continuity gaps
Sturman, 2020a [35] Australia	‘Genuine doctor care’: Perspectives on general practice and community- based care of Australian men experiencing homelessness	To explore the experiences and attitudes of homeless men regarding community-based healthcare, and general practice in particular, To identify the potential areas for improvement	PEH *N* = 20 Ozcare residential hostel for homeless men	Community based healthcare—in particular general practice Primary Healthcare	Focus groups *n* = 5	Three key themes identified important aspects of client experiences: the relative invisibility and low salience of general practice compared to hospital-based emergency and inpatient services; discontinuity within community- based healthcare and across transitions between community-based and other healthcare; and inconsistent and unsatisfactory general practitioner responses to physical and psychological pain. Less prominent, themes were: generalist medical expertise and perceived conflicts of interest for health and social care providers
Sturman, 2020b [36] Australia	‘I just hope they take it seriously’: homeless men talk about their health care	To improve system responsiveness and patient outcomes, the perspectives of marginalised groups need to be understood—Men who experience homelessness	PEH *N* = 20 Ozcare residential hostel for homeless men, Brisbane, Australia	No specific health service exposure	Focus groups *n* = 5	Participants in all groups expressed gratitude for health care provided in both hospital and community sectors for life threatening physical illness and trauma. However, negative experiences with health care were commonly reported.: dismissive care, care fragmentation, inconsistent medical management of pain and inadequate acknowledgement of psychological distress. These four themes relate to difficulty securing an effective ‘ticket of entry’ to health care
Ungpakorn, 2020 [54], UK	Health-related street outreach: exploring the perceptions of homeless people with experience of sleeping rough	To understand how health‐related street outreach is perceived by homeless people with experience of sleeping rough	PEH *N* = 10 Drop‐in centres	Health-related street outreach Primary Healthcare	F2F Semi-structured interviews	Health‐related street outreach was perceived as being able to offer a human connection that reduced the sense of isolation and exclusion commonly experienced on the street. People with experience of sleeping rough felt it could overcome access barriers and provide a bridge to healthcare services. Crucially the right approach was defined by participants in terms of location, timing, the outreach team, and the verbal and non‐verbal styles used by outreach workers. Three main themes were found during the data analysis: A human connection, Street outreach as a bridge and the right approach
Varley, 2020 [55] US	Exploring quality of primary care for patients who experience homelessness and the clinicians who service them: what are their aspirations?	To develop and evaluate an effective model of patient-centred, high-quality, homeless-focused primary care, our team explored key domains of primary care that may be important to patients	PEH *N* = 36 VA and non-VA clinics Health Care for the Homeless Programs	Primary Healthcare	Semi-structured interviews (in person and over the phone)	Template analysis revealed factors important to this population. These included stigma, respect, and perspectives on patient control of medical decision-making in regard to both pain and addiction
Warren, 2021 [56], UK	Developing an Embedded Nursing Service within a Homeless Shelter: Client’s Perspectives	To illuminate the diversity and complexity of healthcare needs of homeless people, as well as offers a unique insight into the service user’s perception of the service	PEH *N* = 6 An embedded nursing service within the homeless shelter	An embedded nursing service within the homeless shelter Primary Healthcare	Semi-structured interviews	Three broad themes were identified in this study; (1): impact of previous healthcare experiences; (2) benefits of embedding healthcare within the shelter and; (3) future service development. Experiences of discrimination, stigma and social exclusion impact how people experiencing homelessness view and access health services and how they perceive the nurse-led clinic within the homeless shelter they use. Narratives of those who use it give testament to the value of embedding nurse-led services within homeless support
Whitley, 2013 [57], US	Fear and loathing in New England: examining the health-care perspectives of homeless people in rural areas	To elicit health-care beliefs, and examine overall health experience among a sample of current or recently homeless people in rural New Hampshire	PEH *N* = 13 Tri-County Community Action Programs	No specific health service exposure	Interviews	Despite a massive burden of disease and illness, many participants reported a strong and abiding aversion to doctors, hospitals and professional health care In contrast to this dislike of professional medical care, participants spoke more fondly of other organisations that provided social services, for example churches and homeless organisations. Three main themes discussed were: Aversion to professional health care, favourable attitudes to social and voluntary supports and popular health care: self-care and self-help
Wise, 2013 [58] US	Hearing the Silent Voices: Narratives of Health Care and Homelessness	To gain understanding about the homeless person’s experience of health care	PEH *N* = 11 Homeless ministry in Knoxville	No specific health service exposure	Interviews	The four polar themes that emerged from the analysis—same/different, fair/unfair, freedom/barriers, and choice/no choice—highlighted the great divide between the health care experiences of those with a home and those without. All participants made it clear that their experience in the healthcare system could be understood only when their world was understood

*PEH *People Experiencing Homelessness, *FTF *Face-to-face, *QI *Quality Improvement, *VA *Veteran Affairs

^a^Purkey 2019 survey excluded from analysis as no patient experience measures

**Table 5:** Mixed methods

Author, year Country	Title	Objective	Population/Setting	Exposure	Patient Experience Measure	Key findings related to patient experience measures and/or outcomes
Bennet-Daly, 2021 [59] Australia	Development and Initial Evaluation of a Nurse-Led Healthcare Clinic for Homeless and At-Risk Populations in Tasmania, Australia: A Collaborative Initiative	To examine barriers to healthcare access amongst individuals who experience homelessness, client and staff perceptions of the MHNC services and explored opportunities for service expansion	PEH administrative case reviews *n* = 174 PEH Interviews *n* = 10 Mission Health Nurse-led Clinic (MHNC)	Mission Health Nurse-led Clinic Primary Healthcare	FTF interviews	The MHNC services were reported to be highly appreciated by all clients. Three themes emerged from the findings: personal vulnerability (client level), disconnectedness (system level) and the acceptability of the MHNC (service level). Subthemes of hardship and adversity, homelessness, lack of empowerment, lived experiences and wellbeing, gaps in or between services, social stigma and societal expectations, expense of health services, rapport and trust, continuity of care, drop-in and fee-free service, client advocacy and health promotion were identified. An expansion of services, extra operating hours and maintenance of flexible appointments were suggested as a means to increase engagement for improved health outcomes. A collaborative model of nurse-led healthcare service can mitigate the challenges of disconnectedness with other primary healthcare services, such as improved access and equity
Chrystal, 2015 [38], US	Experience of primary care among homeless individuals with mental health conditions	To inform healthcare improvement, studied predictors of favourable primary care experience among homeless persons with mental health conditions treated at sites that varied in degree of homeless-specific service tailoring	PEH with mental health conditions *N* = 366 Primary Care Clinics (VA and Non-VA, tailored and non-tailored)	Comparison of homeless specific tailored versus non-tailored Primary Care Clinics Primary Healthcare	The Primary Care Quality-Homeless (PCQ-H) questionnaire	Significant predictors of a positive experience included: a site offering tailored service design, perceived choice among providers, currently domiciled status. For persons with severe psychiatric symptoms, a homeless-tailored service design was significantly associated with a more favourable primary care experience
Greysen, 2012 [28], US	Understanding Transitions in Care from Hospital to Homeless Shelter: a Mixed-Methods, Community-Based Participatory Approach	To understand patients’ experiences of transitions from hospital to a homeless shelter, and determine aspects of these experiences associated with perceived quality of these transitions	PEH *N* = 98 Columbus House (Homeless shelter)	Yale-New Haven Hospital (YNHH) Acute Care	F2F semi-structured interviews Survey instrument^**a**^	Patients perceived an overall lack of coordination between the hospital and shelter at the time of discharge. Expectations of suboptimal coordination exacerbate delays in seeking care. Patients made three recommendations for improvement: 1) Hospital providers should consider housing a health concern; 2) Hospital and shelter providers should communicate during discharge planning; 3) Discharge planning should include safe transportation. 44% of participants reported that housing status was assessed and 42% reported that transportation was discussed. 27% reported discharge occurred after dark; 11% reported staying on the streets with no shelter on first night after discharge

*PEH *People Experiencing Homelessness, *FTF *Face-to-face, *VA *Veteran Affairs

^a^Greysen 2012 survey excluded from analysis as no patient experience measures

**Table 6:** Quantitative studies

Author, year Country	Title	Objective	Population/Setting	Exposure	Patient Experience Measure	Key findings related to patient experience measures and/or outcomes
Behl-Chadha, 2017 [37], US	Comparison of Patient Experience between a Practice for Homeless Patients and Other Practices Engaged in a Patient—Centered Medical Home Initiative	To understand how patient experience differs between a PCMH demonstration practice designed for homeless people in Massachusetts and other practices participating in the same state-wide initiative	PEH *n* = 194 Comparison practice patients *n* = 1,868 Boston Health Care for the Homeless Program (BHCHP)	Comparison with 34 non-rural practices from The Massachusetts Patient-Centered Medical Home Initiative (MA PCMHI) Primary Healthcare	Patient experience surveys were adapted from the Consumer Assessment of Healthcare Providers and Systems Clinician and Group (CG- CAHPS) survey	BHCHP patients gave higher ratings than patients from comparison practices for *Self- management support* (74 vs. 64; *p* < .001) and *Behavioral health integration* (74 vs. 66; *p* < .01). There were no statistically significant differences between the two groups on *Overall rating of the provider, Providers discuss medication decisions* and *Follow-up on test results.* BHCHP scored lower than comparison practices on *How well providers communicate with patient*s (82 vs. 90; *p* < .001), *Helpful, courteous and respectful office staff* (76 vs. 85; *p* < .001), and *Getting timely appointments, care, and information* (69 vs. 79; *p* < .001)
Gabrielian, 2021 [39] US	Enhancing Primary Care Experiences for Homeless Patients with Serious Mental Illness: Results from a National Survey	To assess if primary care teams tailored for homeless patients (Homeless-Patient Aligned Care Teams (H-PACTs) provide superior experiences than mainstream primary care and to explore whether integrated behavioural health and social services are associated with favourable experiences	PEH *N* = 1,095 Veterans Administration integrated healthcare system	Comparison of High integration H-PACTs (3–4 embedded services) to Low integration H-PACTs (0–2 embedded services) and to mainstream services Primary Healthcare	The Primary Care Quality-Homeless (PCQ-H) questionnaire	Homeless-tailored clinics with highly integrated services were associated with better care experiences among PEH with SMI. These observational data suggest that tailored primary care with integrated services may improve care perceptions among complex patients. In all 4 domains (Access/Coordination, Patient-Clinician Relationships, Cooperation, and Homeless-Specific Needs) high integration H-PACT respondents were significantly (*P* < .05) more likely than their mainstream peers to report favourable and/or less likely to report unfavourable experiences. Highly integrated clinics with embedded services were associated with favourable perceptions of clinic access/ coordination. Behavioral health services (eg. addiction services) were not associated with more favourable experiences
Jones, 2017 [41], US	A National Evaluations of homeless and non homeless veterans’ experience with primary care	To compare the primary care experiences of homeless and non-homeless Veterans with Mental Health and/or Substance Use Disorders (MHSUDs) receiving care in the Veterans Health Administration’s medical home environment, called Patient Aligned Care Teams (PACTs)	PEH *N* = 4,605 Non-homeless Veterans *n* = 63,061 In the final weighted sample, 9.2% of Veteran respondents with MHSUDs were homeless. Veterans Health Administration (VHA), PACTs, National database	Comparison of homeless veterans and non-homeless veterans Primary Healthcare	PCMH-SHEP survey—Based on the Consumer Assessment of Healthcare Providers and Systems (CAHPS) PCMH Survey (version 2.0)	After controlling for sociodemographic and clinical characteristics Homeless Veterans reported more negative and fewer positive experiences with communication (Risk Difference (RD) = 1.74 and -3.90, respectively). Homeless Veterans also reported more negative provider ratings (RD = 1.95), comprehensiveness (RD = 2.84), care coordination (RD = 2.35), and medication decision-making (RD = 2.08). After adjusting for covariates, homeless Veterans also reported more negative experiences with self-management support (RD = 2.30). No significant differences were observed in experiences with access or office staff helpfulness/courtesy after adjusting for covariates
Jones, 2021 [40], US	Perceptions of care coordination among homeless veterans receiving medical care in the veterans health administration and a community care setting results from a national survey	To evaluate community care use and satisfaction, and compare perceptions of care coordination among Veterans with homeless experience using VHA services and community care to those using VHA services without community care	PEH *N* = 4777 PEH using VHA services and community care *n* = 1,325 (26.7%) PEH using VHA services without community care *n* = 3,452 (73.3%) Veterans Health Administration (VHA), Community Care Veterans Choice Program	Comparison of homeless veterans using VHA services and community care and those using VHA services without community care Primary Healthcare	The Primary Care Quality-Homeless (PCQ-H) survey (Experiences with Access/Care Coordination) Self-reported use of community care Satisfaction with Community Care	Of 4777 respondents, 1325 (26.7%) reported using community care; most of this subsample affirmed satisfaction with the community care they received (83%) and its timeliness (75%). Satisfaction with community care was lower among patients with travel barriers, psychological distress, and less social support Compared to those using the Veterans Health Administration(VHA) services without community care, Veterans using VHA services and community care were more likely to report unfavorable access/coordination experiences ([OR] = 1.34, CI = 1.15–1.57). This included hassles following referral (OR = 1.37, CI = 1.14–1.65) and perceived delays in receiving health care (OR = 1.38, CI = 1.19–1.61)
Kertesz, 2013 [43], US	Comparing homeless persons’ care experiences in tailored versus non tailored primary care programs	To compare homeless patients’ experiences of care in health care organisations that differed in their degree of primary care design service tailoring	PEH *N *= 601 VA mainstream Primary Healthcare settings, homeless -tailored VA PHC clinic, and a tailored non-VA Health Care for the Homeless Program	Comparison of VA mainstream PHC settings, homeless-tailored VA PHC clinic and a tailored non-VA Health Care for the Homeless Program Primary Healthcare	The Primary Care Quality—Homeless (PCQ-H) Survey (mail and telephone contact)	Tailored primary care service design was associated with a superior service experience for patients who experienced homelessness. Scores at the tailored non-VA site were higher (reflecting more positive experience with care) than those at the 3 mainstream VA sites. After adjusting for patient characteristics, differences remained significant for the relationship (*P* < .001) and cooperation (*P* = .005) subscales, whereas they fell short of statistical significance in the case of access or coordination (*P* = .055) and homeless-specific needs (*P* = .21). There were 1.5- to threefold increased odds of an unfavourable experience in the domains of the patient–clinician relationship, cooperation, and access or coordination for the mainstream VA sites compared with the tailored non-VA site; the tailored VA site attained intermediate results
Kertesz, 2021 [42], US	Comparison of patient experience between primary care settings tailored for homeless clientele and mainstream care settings	To examine whether homeless-tailored primary care programs offer a superior patient experience compared to non-tailored (“mainstream”) programs overall, and for highly vulnerable patients	PEH *N* = 5766 VA, 26 National Medical Centers	Comparison of homeless-tailored primary care (H-PACT) and mainstream primary care (PACT) Primary Healthcare	Primary Care Quality—Homeless (PCQ-H) Survey	H-PACTs outscored mainstream PACTs on all scales (all *p* < 0.001). Unfavourable care experiences were more common in mainstream PACTs compared to H-PACTs, with adjusted risk differences of 11.9% (95% CI = 6.3–17.4), 12.6% (6.2–19.1), 11.7% (6.0–17.3), and 12.6% (6.2–19.1) for Relationship, Cooperation, Access/Coordination, and Homeless-Specific Needs, respectively. For the Relationship and Cooperation scales, H-PACTs were associated with a greater reduction in unfavourable experience for patients with ≥ 2 vulnerabilities versus ≤ 1 (interaction *p* < 0.0001)
Vellozzi-Averhoff, 2021 [32] US	Disparities in communication among the inpatient homeless population at a safety-net hospital	To determine whether the homeless population experiences disparities in care and communication during inpatient hospitalisations in a safety-net hospital	PEH *n* = 33 Non-homeless *n* = 79 University affiliated urban safety-net hospital	Hospital-Based Care	Modified Hospital Consumer Assessment of Healthcare Providers and Systems (HCAHPS) survey	Homeless participants trended toward poorer ratings for all HCAHPS subscales, however Differences between PHQ-2 positive scores between the two cohorts did not reach significance

*PEH* People Experiencing Homelessness, *FTF* Face-to-face, *VA* Veteran Affairs, *VHA* Veteran Health Administration

Most (*n* = 24) studies did not detail how PEH were identified. Of those that did, five studies used the International Classification of Diseases (ICD)-9 or ICD-10 code to identify PEH [32, 38, 39, 41, 43], one study used Veterans Health Administration administrative records [42], one study used clerical staff to identify PEH by accommodation status on arrival to ED [49] and one study used staff to recruit participants as per the homeless definition in the Stewart B. McKinney Homeless Assistance Act [57]. Sample sizes of PEH ranged from five [52] to 68 [31] for qualitative studies and 33 [32] to 5,766 [42] for quantitative studies. Three of the studies from the US examined homeless veterans [39–41].

### Qualitative study results

This section describes firstly the results from the 24 qualitative studies reviewed including the frequency of domains and core values and key themes (Table 7).

**Table 7:** IOM Domains & core values

		**IOM Domains**						**Core Values**						
**No. of artics**	**Author**	**Safety**	**Effective**	**Person-centered**	**Accessible and timely**	**Efficient**	**Equitable**	**Dignity and respect**		**Kindness with compassion**	**Holisitic**	**Partnership and co-production**	**Communication**	**Total domains and valyes**
1	Bennett-Daly, 2021	✔			✔		✔	✔		✔	✔	✔		**7**
2	Davis, 2012	✔	✔	✔	✔	✔				✔	✔		✔	**8**
3	Greysen, 2012	✔				✔					✔		✔	**4**
4	Gunner, 2019	✔	✔	✔	✔	✔	✔	✔		✔	✔	✔	✔	**11**
5	Henderson, 2022	✔	✔	✔	✔	✔	✔	✔		✔	✔	✔		**10**
6	Hirst, 2021	✔		✔	✔		✔	✔		✔	✔	✔		**8**
7	Lamanna, 2018			✔	✔	✔		✔		✔	✔	✔		**7**
8	McCallum, 2020	✔		✔	✔		✔	✔		✔	✔		✔	**8**
9	Meehan, 2023				✔		✔	✔		✔	✔			**5**
10	Moore, 2011			✔			✔	✔		✔	✔		✔	**6**
11	Moore-Nadler, 2020		✔		✔	✔	✔	✔		✔				**6**
12	Nicholas. 2016	✔	✔	✔	✔	✔	✔	✔		✔	✔	✔	✔	**11**
13	Paradis-Gagne, 2023			✔	✔		✔	✔		✔	✔	✔	✔	**8**
14	Purkey, 2019	✔		✔	✔		✔	✔		✔		✔		**7**
15	Saharan, 2021	✔		✔	✔	✔	✔	✔		✔	✔	✔		**9**
16	Steward, 2016	✔	✔	✔	✔	✔					✔	✔	✔	**8**
17	Strange, 2018	✔		✔	✔	✔		✔		✔		✔	✔	**8**
18	Sturman, 2020a	✔	✔	✔	✔	✔		✔		✔		✔	✔	**9**
19	Sturman, 2020b	✔	✔		✔	✔		✔		✔		✔	✔	**8**
20	Ungpakorn, 2020	✔		✔	✔		✔	✔		✔		✔	✔	**8**
21	Varley, 2020	✔	✔	✔	✔	✔	✔	✔		✔	✔	✔	✔	**11**
22	Warren, 2021			✔	✔		✔	✔		✔	✔	✔		**7**
23	Whitley, 2013		✔	✔				✔		✔		✔		**5**
24	Wise, 2013	✔			✔	✔	✔						✔	**5**
	**Frequency**	17	10	18	21	14	16	20		21	16	17	14	

### Domains

The most frequent domain identified was ‘accessible and timely’, appearing across 21 of the 24 included articles. Accessible and timely incorporates timeliness and access to care, the availability and flexibility of services, and geographic and financial accessibility. The most common patient experiences reported within this domain were timeliness of treatment [31, 33–36, 59], flexibility and convenience of services [45, 46, 51, 56] (especially those offering co-located [46, 53–55] or drop-in services [34, 45, 53, 56, 59]), the physical and organisational environment [46], and the location of services [33, 34, 53–55].

The domain of ‘person-centred’ was cited in 18 of the qualitative articles. Person-centred care incorporates the diverse experiences of individuals and their specific needs and priorities. Repeatedly, participants valued services that recognised the complexities relating to homelessness and which tailored services to meet their needs [33, 34, 52, 53]. PEH greatly appreciated the social capital gained from health services, in particular positive social interaction, shared norms and decreased feelings of isolation [30, 46, 53]. Participants described experiences of institutional practices inconsistent with patient-centred care [31, 51, 54], rushed treatment [35, 45, 56] and a lack of awareness of PEH and the complexity of their healthcare needs [28, 29, 45].

The domain of ‘Safety’ encompassed physical and psychological safety, trauma-informed care and health accountability, and was raised in 17 of the 23 qualitative articles. Safety for PEH was most often discussed in relation to the physical environment of health services [46, 53], discharge practices [28, 31, 53] and rapport with healthcare personnel [30, 54, 59]. However, cases of physical assault by security personnel [36], mechanical restraint [31], and stigma from health professionals had a negative impact on their willingness to access services [33, 45, 52]. While accountability was considered an important characteristic [44], in only one paper did PEH expressed concerns that health services were not accountable [29].

The ‘equitable’ refers to care being fair and impartial regardless of individual traits or circumstances, and was domain identified in 16 of the articles reviewed. Examples of equity arose most commonly regarding prejudicial care [29, 31, 33, 48, 51, 52, 55, 56, 58] and healthcare service exclusions [29, 45, 49, 50], with one example reported describing a possible violation of the Emergency Medical Treatment and Labor Act [50].

‘Efficient’ appeared in 14 articles, referring predominantly to the navigation and coordination of services across providers and settings. A common issue raised was the struggle PEH experienced navigating healthcare systems [30, 33, 34, 45, 50, 52, 58]. Instances varied from difficulty understanding health systems [45, 52, 58] to the inherent complexity of referral systems [50] and paperwork [58]. A lack of healthcare insurance also exacerbated difficulties with service navigation in a US-based paper [52]. Case workers or navigators who could help facilitate the navigation of services were seen as critical enablers [30, 33, 34, 36, 52]. In circumstances where PEH accessed ‘navigational aids’, greater usage and higher levels of satisfaction with health services were reported [52]. Although in a paper by Steward (2016), coordination ranked as one of the four most important characteristics of homeless healthcare service provision [44], multiple negative accounts were reported describing episodes of fragmented care [35, 36], wasted time [33], service gaps [31], a lack of coordination between services [28] and problematic relationships with interdisciplinary teams [33]. In one study, 44% of PEH delaying healthcare attributed this to previous experiences and concerns that they would not receive appropriate healthcare [28]. Positive experiences recounted the arrangement of hospital admissions [53], seamless system navigation [31], centralised care coordination that reduced the need for ED presentations [33], and effective coordination and timely referrals that alleviated service user stress [34].

The ‘effective’ domain that pertains to care following evidence-based guidelines and standard operating procedures was the least frequently articulated domain in the qualitative papers reviewed (10 citations), with Steward et al. (2016), independently reporting that PEH highly prioritised ‘evidence-based decision making’ [44]. On the few occasions the effective domain was highlighted in the literature, it involved treatment based on individual biases and stereotypes [33, 50], inconsistent discharge practices [45] and pain management regimens [35, 36].

### Core values

The core values of ‘dignity and respect’ and ‘kindness with compassion’ were detected in 20 articles. Key features of these core values included the acceptance and respect of all views in decision-making, warm and welcoming clinicians and the provision of empathetic and non-judgmental care. For PEH, respectful [46, 49, 51, 53–55] and non-judgmental care was important [29, 47, 49, 51, 53, 56, 59]. Positive experiences were characterised by welcoming and approachable staff [29, 34, 52, 59], human connection [54], being known by name [46], rapport and trust [29, 54, 59], compassionate care [29], the preservation of anonymity [51] and confidentiality [55], and recovery-oriented approaches that led them to feel included in society [54]. Yet there were occasions where PEH perceived health professionals as uncaring [31], demeaning [57], dismissive [35, 36, 50] and judgemental [31, 35, 50, 52, 56, 57]. Many PEH reported experiences of stigmatisation when accessing healthcare [29, 31, 45, 53, 55, 56, 59]. Furthermore, some PEH held concerns of prejudicial information in medical records influencing the provision of medical care [35, 36, 55]. Negative experiences created power dynamics [33], strained relationships [51], adversely impacted care [33], were associated with a loss of self-confidence [56], and contributed to a reluctance to engage healthcare services in the future [29, 31, 33, 45, 51, 52, 56]. In contrast, positive experiences encouraged health-seeking behaviour and service engagement [34, 45], upheld dignity [29] and decreased feelings of shame among PEH [30].

‘Partnership and co-production’ was raised in 17 of the articles reviewed. This value refers to how PEH can be engaged and active partners in designing healthcare and the delivery of services. Integral to this core value is patients at the centre of control. PEH placed high value on decision-making [55]; they wanted to set their own agendas, be asked what they needed, and be allowed to decide for themselves [54]. In one circumstance, PEH perceived that they were full partners with a sense of control over their care, even reporting freedom and an increased choice to change health providers [33]. However, the characteristic of patients as a ‘source of control’ in PHC was ranked relatively low (10 out of 16) [44], and some patients were ambivalent about control when discussing pain and controlled substances [55]. PEH appreciated continuity of care and being able to see the same providers consistently [55, 59]. Although care could be inconsistent or suboptimal [45], overly prescriptive [52], or constrained by distrust in healthcare professionals [29, 46, 52, 53], there was acknowledgement that experiences and engagement improved with time and upon building therapeutic relationships. [35, 36, 46, 53, 56]

The ‘Holistic’ core value was identified in 16 articles. It refers to care that is integrated and addresses physical, emotional, social, spiritual and mental wellbeing. Several articles highlighted failures to provide holistic care [31, 33, 45, 49]. In one article, PEH reported a lack of inquiry into their housing status, with only 44% being assessed during an acute care episode [28]. On the contrary some publications demonstrated endeavours to provide holistic care [30, 46] and the positive effects of treating patients as individuals with personal needs and goals [34].

The core value of ‘communication’ incorporates communication methods, information sharing and awareness of services and was raised in 14 articles. On several occasions, it was apparent that PEH were unaware of their health entitlements and existing outreach, after-hours or primary health care services due to a lack of signposting and communication [35, 45, 49, 51, 54, 55]. This insufficiency in communication extended beyond the realm of access and navigation of services and into treatment care and follow-up, and PEH reported circumstances where communication with kin did not occur [31], unclear post discharge management plans [53] and a system-level reliance on postal and telecommunication methods that was unsuitable for this cohort [55]. PEH ranked ‘shared knowledge and the free flow of information’ in the top 25th percentile of important characteristics of homeless care [44]. People experiencing homelessness called for greater communication between hospitals and shelters during discharge in an effort to improve the coordination of care [28].

### Quantitative study results

The following section reports on the characteristics, settings and frequency at which domains and core values were reported in surveys across the quantitative papers.

Ten articles were initially identified with survey components [28, 29, 32, 37–43], however two were excluded from the analysis, as the surveys contained no patient experience questions [28, 29]. The remaining eight articles utilised an original or adaptation of one of three patient experience surveys [32, 37–43]. Surveys ranged from having 15 items to 33 items and four to seven scales. Across different scales and their respective questions, the frequency of domains and values identified and authenticated ranged from one to seven, and of the 16 scales reviewed, only six scales were identified to exclusively reflect a single domain or value.

The Primary Care Quality-Homeless (PCQ-H) questionnaire was the most frequent survey tool, appearing in five articles. The Consumer Assessment of Healthcare Providers and Systems Survey (CAHPS) was utilised twice, and one study utilised a modified version of the Hospital Consumer Assessment of Healthcare Providers and Systems (HCAHPS) survey. Each of these three survey tools are discussed below in additional detail. Across all three patient experience surveys, the majority of questions pertained to the ‘person-centred’ and ‘accessible and timely’ domains, followed by the core values of ‘dignity and respect’ and ‘communication’. Together, these four domains and values constitute 67% of survey questions. The fewest questions were dedicated to the domains of ‘effective’ ‘equitable’, ‘holistic’ and ‘partnership and co-production’. The least frequently highlighted domains and values were ‘equitable’ and ‘holistic’, with one out of three surveys detailing corresponding questions. No questions addressed the ‘safety’ domain (See Table 8).

**Table 8:** Patient experience surveys and how they map to IOM domains and core values

	**PCQ-H**	**CG-CAHPS** **and supplemental PCMH items**	**HCAHPS**
**Survey Characteristics**	*33 items,4 scales, & and overall score*	*21-items*^a^ *(13-CAHPS, 8-PCMH items),7 scales, & an overall rating of providers*	*15 items, 5 scales*
**Survey Setting**	*Primary Care*	*Primary Care*	*Hospital-Based Care*
**Validity and Reliability**	*Validated in homeless populations (US)* [60]	*Valid and reliable for US population* [61]*. Not specifically for homeless populations*	*Endorsed by US Agency for Healthcare Research and Quality and National Quality Forum* [62]*. Not specifically for homeless populations*
**Appears In**	*Chrystal (2015), Gabrielian (2021), Jones (2021), Kertesz (2013), Kertesz (2021)*	*Behl-Chadha (2017), Jones (2017)*	*Vellozzi-Averhoff (2021)*
***IOM Domains—n***			
Safety	-	-	-
Effective	1	-	2
Person Centred	8	7	2
Accessible & Timely	11	4^*a*^	2
Efficient	4	1	-
Equitable	2	-	-
**Core Values—n**			
Dignity & Respect	2	2	2
Kindness with Compassion	3	1	-
Holistic	-	3	-
Partnership & co-production	1	1	1
Communication	1	2	3

^a^Jones, 2017 had six-items under the access domain (compared to four-items with Behl-Chadha, 2017), one additional CAHPS item, one additional PCMH item, surveys otherwise utilised similar structured questions throughout CG-CAHPS

#### Primary Care Quality-Homeless (PCQ-H) questionnaire

The PCQ-H was the most comprehensive survey; it covered all domains and values with the exception of ‘safety’ and ‘holistic’. General constructs of the PCQ-H survey were based on IOM publications [25, 63], a card sort exercise [44] and qualitative interviews and focus groups with PEH and homeless care provider experts [60]. The PCQ-H is advantageous, as it is the only survey reviewed that has been specifically developed for and that has had its validity and reliability determined for PEH [60]. This is important because the concerns and needs of PEH differ from those of the general population and may be overlooked in standard survey instruments. The PCQ-H has been specifically designed to account for low literacy comprehension (seventh grade reading level) to ensure understandability [60]. The majority of authors utilised the PCQ-H in its entirety with the exception of Jones (2021), who only utilised the 11-item Access/Coordination scale from the PCQ-H.

#### Consumer Assessment of Healthcare Providers and Systems Clinician and Group (CG-CAHPS)

The CG-CAHPS survey questions identified eight of the 11 IOM domains and core values, with the exception of ‘safety’, ‘effective’ and ‘equitable’. The CAHPS surveys were originally designed to compare service providers and assist consumers in judging health plans [64, 65]; however, the CG-CAHPS was initially developed to measure patient experiences in ambulatory care [61]. Behl-Chada, (2017) and Jones, (2017) both utilised modified surveys based on version 2.0 of the CG-CAHPS, with supplementary Patient-Centered Medical Home (PCMH) items to allow for a more comprehensive assessment of PHC and patient experience. The psychometric properties of the CG-CAHPS have been reported to be acceptable [61].

#### Modified Hospital Consumer Assessment of Healthcare Providers and Systems (HCAHPS) survey

The HCAHPS survey covered just over half of the IOM domains and core values. The standard HCAHPS survey is typically 27 items; however, Vellozzi-Averhoff utilised fewer items in their study [32]. The HCAHPS survey is used among hospitals for inpatient care, which allows for cross-industry service quality comparison. A study on the psychometric properties of HCAHPS raised concerns about the consistency, reliability and validity of multiple-item measures for service quality [66]. Furthermore, the sampling frame utilised for the development of HCAHPS excluded patients who were not discharged ‘home’ based on the premise that these patients were less likely to respond to surveys [67]. As a result, differing responses from PEH may not be reflected within the existing psychometric properties of HCAHPS.

### Summary of results for domains and core values

‘Accessible and timely’ and ‘person-centred’ domains were identified in the literature as the most prominent domain 20 and 18 times in the literature respectively. Moreover in two out of the three patient experience surveys, these two domains were more commonly identified in survey questions. The core values of ‘dignity and respect’, ‘partnership and co-production’ and ‘communication’ were highlighted between 14 and 20 times and were reported across all three surveys. ‘Efficient’ and ‘kindness with compassion’ were highlighted 14 and 20 times, respectively, and both were reported in two surveys. The ‘effective’ domain was described the least in the literature (10 times) but was identified in two surveys. The ‘equitable’ and ‘holistic’ domains were described in 15 articles each; however, ‘equitable’ only appeared in the PCQ-H survey, and ‘holistic’ only appeared in the CAHPS survey. The ‘safety’ domain was cited in 17 articles; however, no survey questions addressed the safety domain.

## Discussion

The most frequent domains and core values that emerged upon review of the literature for PEH healthcare experiences were ‘accessible and timely’, ‘person-centred’, ‘dignity and respect’ and ‘kindness with compassion’. Of the three existing patient experience surveys identified in this field, the PCQ-H most accurately encompasses the findings voiced in the qualitative literature. Less emphasis was found in the patient experience data on ‘communication’, ‘effective’ and ‘efficient’ than seen in the surveys.

### Accessible and timely care

‘Accessible and timely’ care was the domain raised most throughout the literature, and the corresponding survey questions were plentiful and comprehensive. Quantitative survey questions positively encompass the preferences of PEH, giving consideration to wait times, flexibility, convenience and location of services. Historically, PEH have reported significant barriers to accessing care [68], with accessibility highlighted as a top priority by both patients and providers [44]. Given the competing priorities of PEH to meet physiological needs such as shelter and food, it is understandable that health needs are best met in an opportunistic fashion.

### Kindness, compassion, dignity and respect, and person-centred care

Provision of ‘person-centred’ care was highlighted across both types of studies and is considered fundamental to the provision of quality care; it is associated with improved health outcomes and healthcare utilisation [69]. In the literature, PEH consistently valued healthcare that acknowledged homeless-specific needs and tailored services. Perhaps the most fitting question measuring patient experience for ‘person-centred’ care was: *‘My primary care provider makes sure health care decisions fit with the other challenges in my life’* from the PCQ-H.

‘Kindness with compassion’ and ‘dignity and respect’ were the most common values identified in qualitative articles and were moderately represented in patient experience surveys. These core values are central to patient-centred care frameworks [24, 70]. Patient experience of health care services are impacted by a multitude of factors including the behaviours and attitudes of healthcare and professional staff [19]. Respect is considered an essential part of building trusting relationships between PEH and providers [71]. Whilst, kindness with compassion embodied as non-judgemental and empathetic communication can enhance service engagement among PEH [71].

Survey questions relating to the value ‘dignity and respect’ were aimed at authenticating the provision of treatment with courtesy and respect, as well as addressing concerns of anonymity and confidentiality. Whereas survey questions relating to the value ‘kindness and compassion’ affirmed the provision of non-judgemental care non-directly, they did not explicitly ask to what extent were clinicians and non-clinicians empathetic, non-judgmental, warm and welcoming. Questions addressing these values may be less common in surveys due to an inherent difficulty related to quantifying, assessing and measuring abstract ideas such as kindness, respect and dignity.

### Safety, choice, control and holistic care

There is extensive literature documenting the clear relationship between exposure to trauma, poor mental health, and chronic homelessness [72]. For people who have experienced significant trauma, a sense of choice and control over their healthcare is important [73]. In order to engage in effective and meaningful healthcare, individuals must connect and feel safe in the therapeutic relationship.

Primary care is often best placed to develop safe relationships and access community-based services. The ‘safety’ domain was cited in 17 articles; however, no patient experience survey questions explicitly addressed whether providers made them feel safe. Physical and psychological safety was identified by PEH as being important to patient experience. Of note four articles emphasised the importance of trauma-informed care [29, 35, 46, 52] and one article raised cultural safety [29]. Although these topics were highlighted by their respective authors and not raised categorically by PEH themselves.

Within the ‘partnership and coproduction’ value, it is clear that PEH value autonomy around decision-making. Yet the survey questions pertaining to this value allude more so to ‘consultation’ in designing health as opposed to patients as a source of control. There were no survey questions directly related to the strength of therapeutic relationships with healthcare professionals, although some questions assigned to the person-centred domain do reflect the quality of patient-provider relationships. Authors, including researchers with lived experience; observed the notable absence of cultural safety and gender sensitivity in the articles. and gender minorities are disproportionately represented in youth homelessness [74–76] and experience higher rates of trauma [75]. The same minority groups are subject to increased safety risks, and report lower levels of perceived safety when entering shelters and services [75]. Cultural safety is an indigenous-led model of care that was born in New Zealand [77]. There are varying interpretations of cultural safety; however, broadly speaking, definitions encompass power differentials between patient and provider and subsequent associated barriers to clinical effectiveness arising from said power differentials [78]. Distinctive from cultural competency, cultural safety focuses and reflects upon the culture of the clinician, the provision of care, and the healthcare environment [78]. Cultural safety is an important consideration when examining patient experience of PEH, as indigenous populations are overrepresented in homelessness. In 2021, one in five (20.4%) PEH in Australia identified as Aboriginal and or Torres Strait Islander [18]. Similarly, Indigenous and First Nations people from the United States, Canada and New Zealand are overrepresented among homeless populations [79–81]. Cultural safety is intimately linked to health equity and notions of power [78]. For this reason, it is anticipated there would be some cross-over between the safety and equity domains. Despite this potential for overlap, there were no clear examples of discrimination or racism against indigenous PEH cited under equity, and only one article detailed concerns about prejudicial care specific to ethnicity [51]. Similarly, none of the studies reviewed recounted patient experiences specific to sexual and gender minority groups. Although, several articles acknowledged potential limitations of their studies due to gender [28, 34, 35, 51].

An absence of coverage on cultural safety could be reflective in that only a few studies were from Australia and Canada, where cultural safety has gained greater traction within healthcare. Furthermore, patient experience extracted from qualitative articles relating to the ‘safety’ domain may in fact be underrepresented or incomplete. It is possible that safety has not been captured in its entirety, as patients were not explicitly asked about ‘safety’ or these sentiments were captured via the ‘kindness with compassion’ value, which includes warm and approachable clinicians and empathetic and non-judgemental care. Moreover, there is an element of safety that could be assumed or interpreted via other statements from PEH or that is postulated as being built into existing health structures and models of care. Therefore, the inclusion of specific survey questions focused on the ‘safety’ domain may offer a more accurate portrayal of safety for PEH hereafter. Primary experience surveys did not routinely include questions addressing ‘safety’. The exploration of safety as a domain identified by PEH and healthcare providers is a clear gap in the literature that needs to be explored in future studies to develop a shared understanding of this important concept.

Safety, trust, choice, control and collaboration in care are important elements of trauma-informed care [82]. As a result, trauma-informed care crosses over into the ‘holistic’, ‘partnership and co-production’ and ‘kindness with compassion’ values. Consideration should be given to the inclusion of survey questions to measure safety and trauma informed care. *(e.g., do you feel safe when attending healthcare services, considering both your physical well-being and emotional well-being?, how safe and supported do you feel in the healthcare setting in terms of addressing your individual needs and experiences?).*

Survey questions that were exclusively assigned to the ‘holistic’ value were found to be predominantly focused on mental health, and with the exception of one question enquiring about alcohol and substance use, failed to ask patients more broadly about their wellbeing and factors affecting their health. Albeit only one of the three surveys was specifically designed for PEH, none of the surveys had questions pertaining to housing, which for PEH is a critical component to stability, security and health.

### Effective and equitable care

The literature suggests that effectiveness is more likely to be recognised by health providers than by services users [83] and may in part explain why the ‘effective’ domain was infrequently cited. Paucity in articles may be due to scepticism or a lack of relevance to PEH, as evidence-based practice has previously been perceived by PEH as an authoritative top-down approach that reinforces existing modalities of care [84]. Despite the necessity of lived experience to shape the application and interpretation of evidence-based practice, lived experience perspectives and expertise are frequently omitted or poorly utilised [84]. The ‘effective’ domain tended to appear in the literature due to grievances with a lack of adherence to evidence-based guidelines and standard operating procedures and therefore may also reflect concerns about inequity as PEH received treatments based on biases and stereotypes.

The ‘equitable’ domain was not commonly investigated, but it is important because it holds the potential to quell concerns of PEH and expose incidences of prejudicial care, lesser care and or service exclusions. Example questions from the PCQ-H include *(Staff at this place treat some patients worse if they think that they have addiction issues)* and *(At this place, I have sometimes not gotten care because I cannot pay)*. A more general question that purely focuses on prejudicial care may be of benefit due to its broader applicability *(e.g., in your healthcare experiences, to what extent do you feel that care is provided fairly and without bias, regardless of your individual traits or circumstances?).* Potential future survey questions could include whether or not PEH felt their care adhered to required guidelines and standards.

### Efficiency and communication

Despite a common theme pertaining to PEH struggling with the navigation of health care services, only one of the five efficient survey questions somewhat reflected this concern: *(My primary care provider helps to reduce the hassles when I am referred to other services).* No survey questions clearly enquired as to whether PEH received assistance or experienced difficulty navigating services. The remaining ‘efficient’ questions focused on the coordination of services and patient follow-up. The primary discourse for the core value of ‘communication’ related to PEH’s awareness of services and health entitlements. Yet only one survey question clearly represented this notion. Rather, the majority of ‘communication’ survey questions relate to the quality of communication provided by health professionals and whether it was understandable to the individual.

### Future directions and implications for practice

In our analysis, we have made an effort to integrate findings from both qualitative research and surveys. We recognise the intrinsic value, richness and depth provided by qualitative research, which is insightful and more comprehensive than survey findings alone. Given our setting of a hospital service, we hope to gather information that can contribute to the development of patient experience measures, ultimately enhancing the healthcare provided to PEH. To ensure health services are capturing the experience of PEH accurately, surveys and measures of patient experience must be tailored to reflect what matters to PEH when accessing healthcare. For healthcare services that care for PEH, utilising patient experience measures that are adjusted to reflect the complexity of the population increases the acceptability of results and aids fairer comparison across practices [16].

Generic surveys utilised on this cohort are incomplete and inadequately inform patient experience, at times overlooking broader notions of health, and the underlying social determinants of health (such as housing) that may take precedence over outright health needs. Existing surveys do not adequately portray and incorporate all the IOM domains and core values, this is most apparent for ‘safety’, ‘equitable’, and ‘efficient’. Further research is needed to explore whether surveys are asking the correct questions to inform patient experience for PEH. Furthermore, it is possible that themes identified by PEH may also hold value for other marginalised or vulnerable groups who experience healthcare inequities and subsequently poorer health outcomes. In the interests of improving equitable care across healthcare services, themes around personal interactions, as identified in this paper, should be better considered. Further research is needed on whether sufficient patient experience data may be collected via the addition of a subset of questions specific to homelessness to an existing generic survey. Patient experience is complex and multifaceted, and positive measurements in one domain or value may not always translate to quality in other domains or values. Surveys that ask only binary or close-ended responses may fail to capture key patient perspectives and contextual information that is important to PEH [85].

The PCQ-H was most commonly utilised survey tool for PEH in primary research. It was developed with extensive involvement of PEH and has evidence of scientific rigour [60]. Potential further research could include the adaptation and validation of the PCQ-H in other healthcare settings (e.g., hospital based care). This study supports the establishment of the PCQ-H survey as the current gold standard within the primary healthcare setting for PEH. This is indicated by its reflection most closely of themes identified in the qualitative literature.

Challenges exist in routinely collecting patient experience measures in healthcare settings [86, 87]. Whilst existing studies do not specifically examine PEH, the completion of patient experience measures is impacted by social determinant of health [87], and barriers are transferable and applicable to PEH; such as language proficiency, health literacy, technology literacy, cognitive functioning and time constraints [86]. For PEH additional barriers to survey engagement may include: stigma, poor mental health, substance or alcohol abuse, competing priorities, the transient nature of homelessness and high rates of discharging from health services against medical advice. Co-design projects and tailored approaches are required to overcome barriers and optimise the collection of patient experience data [88], in turn assessing and exploring how healthcare organisations can best respond to the findings so that healthcare for PEH can be improved. Possible directions for future research include developing the findings, such as incorporating a Delphi design and further validation studies to refine survey recommendations for these broader healthcare services.

### Strengths and limitations

A strength of this study was the involvement of people with lived experience of homelessness in the design, analysis and interpretation of the papers. The inclusion of people with lived experience provided contextual understanding to the topic, ensured relevance and enhanced authenticity and validity of the study. A further strength of this paper was that it includes both qualitative and quantitative research papers, this allowed for a more comprehensive understanding of the topic and a triangulation of findings to address the scoping aim and secondary objectives. The degree of detail possible by using the extended domains framework assisted in framing the studies in domains that were especially relevant for PEH. This paper also utilised rigorous methodology by having studies reviewed by multiple researchers to reach consensus on domains and core values.

A limitation of this study was the exclusion of specialised health services or disease-focused articles, as there may have been relevant findings from these articles that were applicable to other healthcare settings and PEH. There is a possibility that in determining and allocating a singular primary domain or core value to each survey question, that survey data was simplified and lost some of its breadth. Although the research team undertook a rigorous technique for coding the domains and values, there were instances where more than one domain or value was found to be applicable despite agreed upon definitions. The majority of questions were allocated with consensus.

A significant limitation is that the majority of studies were from the US and four other OECD countries and may not extrapolate to other settings. However, the findings involving domains and core values have the potential to be interpreted universally. OECD countries were chosen so as the findings could be applied to healthcare service development for PEH at the main study site. Due to the large number of articles, grey literature on patient experience was not reviewed. Grey literature may have offered additional value to our study. Lastly, this study was also limited in that exploration of patient experience was confined to existing published research, it is possible that there are elements of patient experience for PEH that are not adequately reflected in the published literature.

## Conclusion

When measures patient experience for PEH, questions pertaining to how the provider treats them, including if they felt respected, if they were shown kindness and compassion, if they were made to feel safe, and if they felt listened to need to be captured alongside how effective and accessible treatment was. This study identifies that ‘kindness and compassion’ questions should be further emphasised when seeking feedback on healthcare experiences from PEH. The domains of ‘safety’, ‘equitable’, and ‘efficiency’ are not adequately represented in existing patient experience surveys.

The PCQ-H was the survey that best reflected the priorities in healthcare provision identified by qualitative analysis and may be suitable to extend to other healthcare settings. Of note, although safety is identified as being a priority for PEH, this was not identified in any of the surveys.

Communication was not a feature of themes recognised as important in qualitative studies, but elements of communication may have been included in other domains. The literature shows that many of the most frequently cited domains and values that PEH expressed to be most important when seeking healthcare were reflected in the three identified survey tools used to varying affect.

### Supplementary Information

**Supplementary Material 1.**

**Supplementary Material 2.**

## Data Availability

The datasets used and/or analysed during the current study are available from the corresponding author on reasonable request.

## Abbreviations

PCMH: Patient-centred medical home
PEH: People experiencing homelessness
