# Supplementary material for: A scoping review examining patient experience and what matters to people experiencing homelessness when seeking healthcare
Source: BMC Health Serv Res. 2024 Apr 20;24:492. doi: 10.1186/s12913-024-10971-8 (PMC11031864; doi:10.1186/s12913-024-10971-8)
Supplement: Supplementary file 1 — Supplementary Material 1. [file 12913_2024_10971_MOESM1_ESM.docx]

**Additional File 1. Search strategy and terms.**

**Supplementary Table 1.** Search strategy per database

**MEDLINE, EMBASE and APA PsychINFO**

Search performed 1^st^ December, 2022

| **No.** | **MEDLINE, EMBASE and APA PsychINFO Search commands** | **Results** |
| --- | --- | --- |
| **1.** | **exp homeless persons/ or exp homeless youth/** | **14418** |
| **2.** | **homeless*.ti,kw.** | **24303** |
| **3.** | **1 or 2** | **29418** |
| **4.** | **exp patient reported outcome measures/ or exp patient outcome assessment/ or exp patient satisfaction/** | **1065996** |
| **5.** | **((patient* or outpatient* or inpatient*) adj2 (experience* or reported* or perspective* or perceive* or feedback* or complaint* or view* or voice* or preference* or satisfaction* or insight*)).ti,ab,kw.** | **938974** |
| **6.** | **4 or 5** | **1813695** |
| **7.** | **3 and 6** | **891** |
| **8.** | **exp health facilities/ or exp health services/ or exp quality of healthcare/** | **18653822** |
| **9.** | **exp Patients** | **3042585** |
| **10.** | **(health* or hospital* or patient* or outpatient* or emergency department*).ti,ab,kw.** | **26256976** |
| **11.** | **8 or 9 or 10** | **33385592** |
| **12.** | **((consumer* or client* or adult* or people*) adj2 (experience* or reported* or perspective* or perceive* or feedback* or complaint* or view* or voice* or preference* or satisfaction* or insight*)).ti,ab,kw.** | **152682** |
| **13.** | **3 and 11 and 12** | **775** |
| **14.** | **exp Health Services Accessibility/** | **216615** |
| **15.** | **Access*.ti,ab,kw.** | **1662641** |
| **16.** | **14 or 15** | **1794064** |
| **17.** | **3 and 12 and 16** | **297** |
| **18.** | **7 or 13 or 16** | **1615** |
| **19.** | **limit 18 to (english language and yr="2008 - 2022")** | **1351** |

**Supplementary Table 2.** Search strategy per database

**CINAHL**

Search performed 1^st^ December, 2022

| **No.** | **CINAHL Search commands** | **Results** |
| --- | --- | --- |
| **S1.** | **(MH "Homeless Persons") OR (MH "Homelessness")** | **10,293** |
| **S2.** | **(MH "Patient-Reported Outcomes+") OR (MH "Outcome Assessment") OR (MH "Patient Satisfaction+")** | **115,467** |
| **S3.** | **("patient*" or "outpatient*" or "inpatient*") N2 ("experience*" or "reported*" or "perspective*" or "perceive*" or "feedback*" or "complaint*" or "view*" or "voice*" or "preference*" or "satisfaction*" or "insight*")** | **203,251** |
| **S4.** | **S2 OR S3** | **248,074** |
| **S5.** | **S1 AND S4** | **261** |
| **S6.** | **(MH "Health Services+") OR (MH "Health Facilities+") OR (MH "Quality of Health Care+")** | **9,643** |
| **S7.** | **(MH "Patients")** | **2,083,305** |
| **S8.** | **"health*" or "hospital*" or "patient*" or "outpatient*" or "emergency department*"** | **4,115,259** |
| **S9.** | **S6 OR S7 OR S8** | **4,624,834** |
| **S10.** | **("consumer*" or "client*" or "adult*" or "people*") N2 ("experience*" or "reported*" or "perspective*" or "perceive*" or "feedback*" or "complaint*" or "view*" or "voice*" or "preference*" or "satisfaction*" or "insight*")** | **51,016** |
| **S11.** | **S1 AND S9 AND S10** | **343** |
| **S12.** | **(MH "Health Services Accessibility+")** | **103,351** |
| **S13.** | **“access*”** | **294,361** |
| **S14.** | **S12 OR S13** | **295,145** |
| **S15.** | **S1 AND S10 and S14** | **146** |
| **S16.** | **S5 OR S11 OR S15** | **592** |
| **S17.** | **limit 14 to (english language and yr="2008 - 2022")** | **486** |
