# Supplementary material for: A scoping review examining patient experience and what matters to people experiencing homelessness when seeking healthcare
Source: BMC Health Serv Res. 2024 Apr 20;24:492. doi: 10.1186/s12913-024-10971-8 (PMC11031864; doi:10.1186/s12913-024-10971-8)
Supplement: Supplementary file 2 — Supplementary Material 2. [file 12913_2024_10971_MOESM2_ESM.docx]

**Additional File 2.**

**Assignment of survey questions to IOM domains and core values for three surveys.**

| **Survey** | | **Primary Care Quality-Homeless (PCQ-H) questionnaire** | |
| --- | --- | --- | --- |
| **Authors** | | **Chrystal, 2015, Gabrielian, 2021, Jones, 2021, Kertesz, 2013 & Kertesz, 2021** | |
| **Subscale** | **no.** | **Question** | **IOM Domain / Core Value** |
| **Patient-Clinician Relationship** | 1 | My PCP never doubts my health needs | **Dignity and respect** |
|  | 2 | My PCP takes my health concerns seriously. | **Person Centered** |
|  | 3 | My PCP makes decisions based on what will truly help me | **Person Centered** |
|  | 4 | I feel my PCP has spent enough time trying to get to know me. | **Person Centered** |
|  | 5 | I can get in touch with my PCP when I need to | **Accessible & Timely** |
|  | 6 | I can get enough of my PCP's time if I need it. | **Accessible & Timely** |
|  | 7 | If my PCP and I were to disagree about something related to my care, we could work it out | **Partnership and co production** |
|  | 8 | My PCP makes sure health care decisions fit with the other challenges in my life | **Person Centered** |
|  | 9 | I worry about whether my PCP has the right skills to take good care of me | **Effective** |
|  | 10 | I can be honest with my PCP if I use drugs or alcohol. | **Kindness with compassion** |
|  | 11 | I worry my PCP might report my health information to the authorities | **Dignity & Respect** |
|  | 12 | Someone from my PCP's office returns my phone calls or pages. | **Accessible and timely** |
|  | 13 | When I need information about my health care, like test results, I can get it easily. | **Accessible and timely** |
|  | 14 | The staff at this place listens to me. | **Kindness and compassion** |
|  | 15 | Staffs at this place treat some patients worse if they think that they have addiction issues | **Equitable** |
| **Cooperation among Clinicians** | 1 | My primary care and other health care providers need to communicate with each other more. | **Efficient** |
|  | 2 | I have been frustrated by lack of communication among my primary care and other health care providers. | **Efficient** |
|  | 3 | My primary care and other health care providers are working together to come up with a plan to meet my needs. | **Efficient** |
| **Access/Coordination** | 1 | My PCP helps to reduce the hassles when I am referred to other services. | **Efficient** |
|  | 2 | I have to wait too long to get the health care services my PCP thinks I need | **Accessible & Timely** |
|  | 3 | At this place, I have sometimes not gotten care because I cannot pay | **Equitable** |
|  | 4 | If I could not get to this place, I think the staff would reach out to try to help me get care. | **Person Centered** |
|  | 5 | If I walk-in to this place without an appointment, I have to wait too long for care | **Accessible & Timely** |
|  | 6 | This place is open at times of the day that are convenient for me. | **Accessible & Timely** |
|  | 7 | This place helps me get care without missing meals or a place to sleep | **Accessible and timely** |
|  | 8 | It is often difficult to get health care at this place | **Accessible & Timely** |
|  | 9 | This place tells me about what services are available. | **Communication** |
|  | 10 | The health care services I need are close to each other | **Accessible & Timely** |
|  | 11 | If my PCP is unavailable there is someone else that can help me | **Accessible & Timely** |
| **Homeless-Specific Needs** | 1 | This place tries to help me with things I might need right away, like food, shelter or clothing. | **Person centred** |
|  | 2 | The people who work at this place seem to like working with people who have been homeless. | **Kindness with Compassion** |
|  | 3 | If I miss an appointment, this place still finds a way to help me. | **Person Centered** |
|  | 4 | At this place, I always have to choose between health care and dealing with other challenges in my life. | **Person Centered** |
| **Primary Care Quality-Homeless Overall Score** | 1 | Provider overall score | **Basic satisfaction measure** |

| **Survey** | | **Modified Consumer Assessment of Healthcare Providers and Systems Clinician and Group Survey (CG-CAHPS)** ^a^ | |
| --- | --- | --- | --- |
| **Authors** | | **Behl-Chadha, 2017 & Jones, 2017** | |
| **Subscale** | **no.** | **Question** | **IOM Domain / Core Value** |
| **Getting timely appointments, care, and information** | 1 | *How often got appointment as soon as needed for urgent care when phoned provider’s office?* | **Accessible & Timely** |
|  | 2 | *How often got appointment as soon as needed for check up or routine care?* | **Accessible & Timely** |
|  | 3 | *How often got answers to medical questions on the same day when phoned during regular office hours?* | **Accessible & Timely** |
|  | 4 | *How often got answer as soon as needed when phoned after regular office hours?* | **Accessible & Timely** |
| **How well providers communicate with patients** | 1 | How often provider showed respect for what you had to say? | **Dignity & Respect** |
|  | 2 | How often provider spent enough time with you? | **Person centered** |
|  | 3 | How often provider explained things in a way that was easy to understand? | **Communication** |
|  | 4 | How often provider listened careful? | **Person centred** |
|  | 5 | How often provider gave easy to understand information about health questions or concerns? | **Communication** |
|  | 6 | How often provider seemed to know the important information about your medical history? | **Person centred** |
| **Helpful, courteous and respectful office staff** | 1 | How often were office staff as helpful as they should be? | **Kindness with compassion** |
|  | 2 | How often office staff treated you with courtesy and respect? | **Dignity & Respect** |
| **Follow-up on test results** | 1 | How often did someone in this provider’s office follow-up to give results about blood test, xray, or other test? | **Efficient** |
| **Providers pay attention to your mental or emotional health** | 1 | Anyone in provider’s office asked if patient had felt sad, empty, or depressed? | **Holistic** |
|  | 2 | Anyone in provider’s office talked about worrying/ stressful aspects of patient’s life? | **Holistic** |
|  | 3 | Anyone in provider’s office talked about personal problem, family problem, alcohol use, drug use, or a mental or emotional illness? | **Holistic** |
| **Providers support you in taking care of your own health** | 1 | Anyone in provider’s office asked if there were things that made it hard for patient to take care of health? | **Person centred** |
|  | 2 | Anyone in provider’s office talked with patient about specific health goal? | **Person Centered** |
| **Providers discuss medication decisions** | 1 | Provider talked to patient about reasons patient might want to take medicine? | **Person Centered** |
|  | 2 | Provider talked to patient about reasons patient might not want to take medicine? | **Person Centered** |
|  | 3 | Provider asked what patient thought was best for patient? | **Partnership and co-production** |
| **Patient’s rating of the provider** | 1 | Rate this provider from 0 (worst) to 10 (best) | **Basic satisfaction measure** |
| ^a^ Questions analyzed were taken from Behl-Chadha, 2017 who utilised a Modified Consumer Assessment of Healthcare Providers and systems (CAHPS), measures based on core CAHPS items and on supplemental patient-centered medical home items designed for the CG-CAHPS survey. Questions utilised in Jones, 2017 were not analysed. | | | |

| **Survey** | | **Modified Hospital Consumer Assessment of Healthcare Providers and Systems (HCAHPS) survey** ^a^ | |
| --- | --- | --- | --- |
| **Authors** | | **Vellozzi-Averhoff, 2021** | |
| **Subscale** | **no.** | **Question** | **IOM Domain / Core Value** |
| **Nursing Communication** | 1 | During this hospital stay, how often did nurses treat you with courtesy and respect? | **Dignity & Respect** |
|  | 2 | During this hospital stay, how often did nurses listen carefully to you? | **Person centred** |
|  | 3 | During this hospital stay, how often did nurses explain things in a way you could understand? | **Communication** |
|  | 4 | During this hospital stay, after you pressed the call button, how often did you receive help as soon as you wanted it? | **Accessible & Timely** |
| **Physician Communication** | 1 | During this hospital stay, how often did doctors treat you with courtesy and respect? | **Dignity & Respect** |
|  | 2 | During this hospital stay, how often did doctors listen carefully to you? | **Person centred** |
|  | 3 | During this hospital stay, how often did doctors explain things in a way you could understand? | **Communication** |
| **Responsiveness** | 1 | How often did you get help in getting to the bathroom or in using a bed-pan as soon as you wanted? | **Accessible & Timely** |
| **Pain Management** | 1 | During this hospital stay, how often did the healthcare team talk with you about how much pain you had? | **Effective** |
|  | 2 | During this hospital stay, how often did the healthcare team talk with you about how to treat your pain? | **Partnership and co-production** |
| **Communication about Medications** | 1 | Before giving you any new medicine, how often did your healthcare team tell you what the medicine was for? | **Effective** |
|  | 2 | Before giving you any new medicine, how often did hospital staff describe possible side effects in a way you could understand? | **Communication** |
| **^a^** The standard HCAHPS Survey is 27-items, Vellozzi-Averhoff  utilised 15 of the 27-items, however only 13-items are documented in the published article. | | | |
